# Multifunctional Tannic Acid-Alendronate Nanocomplexes with Antioxidant, Anti-Inflammatory, and Osteogenic Potency

**DOI:** 10.3390/nano11071812

**Published:** 2021-07-13

**Authors:** Somang Choi, Han-Saem Jo, Heegyeong Song, Hak-Jun Kim, Jong-Keon Oh, Jae-Woo Cho, Kyeongsoon Park, Sung-Eun Kim

**Affiliations:** 1 Department of Orthopedic Surgery and Nano-Based Disease Control Institute, Korea University Guro Hospital, #148, Gurodong-ro, Guro-gu, Seoul 08308, Korea; chlthakd1029@naver.com (S.C.); luchiatkfkd@naver.com (H.-S.J.); dakjul@korea.ac.kr (H.-J.K.); jkoh@korea.ac.kr (J.-K.O.); 2 Department of Systems Biotechnology, Chung-Ang University, Anseong 17546, Korea; island6231@gmail.com

**Keywords:** tannic acid (TA), alendronate (ALN), nanocomplexes (NPXs), ROS effect, inflammation control, osteogenesis acceleration

## Abstract

In the current study, we fabricated tannic acid-alendronate (TA-ALN) nanocomplexes (NPXs) via self-assembly. These TA-ALNs were characterized by dynamic light scattering, zeta potential, transmission electron microscopy, and FT-IR spectroscopy. The TA-ALNs were evaluated for antioxidant, anti-inflammatory, and osteogenesis-accelerating abilities in osteoblast-like cells (MC3T3-E1 cells). All TA-ALNs displayed nano-sized beads that were circular in form. Treatment with TA-ALN (1:0.1) efficiently removed reactive oxygen species in cells and protected osteoblast-like cells from toxic hydrogen peroxide conditions. Moreover, TA-ALN (1:0.1) could markedly decrease the mRNA levels of pro-inflammatory mediators in lipopolysaccharide-stimulated cells. Furthermore, cells treated with TA-ALN (1:1) exhibited not only significantly greater alkaline phosphatase activity and calcium collection, but also outstandingly higher mRNA levels of osteogenesis-related elements such as collagen type I and osteocalcin. These outcomes indicate that the prepared TA-ALNs are excellent for antioxidant, anti-inflammatory, and osteogenic acceleration. Accordingly, TA-ALN can be used latently for bone renovation and regeneration in people with bone fractures, diseases, or disorders.

## 1. Introduction

Nanotechnology is an emerging area that has been extensively present in our daily lives over the past decades in aspects related to energy, the environment and medicine. In the medical field, nanometer-scale nanomaterials provide new tools to systematically characterize, manipulate, and explore diseases using imaging and diagnostic programs [1].

Nanomaterials act as vectors, delivering drugs or therapeutics to obtain better treatment results. Bone diseases result from a variety of skeletal-related disorders including trauma, osteomyelitis, osteosarcoma, and bone infections, which cause major mobility impediments and mortality to human beings [2,3,4]. As previously reviewed, to safely and efficiently treat bone diseases, nano-based materials have been used as drug delivery systems including hydroxyapatite nanoparticles, nano-gel, polysaccharide-based nanoparticles, mesoporous silica nanoparticles, titanium nanotubes, calcium nanoparticles, polyurethane nano-micelles, and biodegradable polymeric nanoparticles [5]. These systems have great advantages in terms of prolong drug half-lives, targeting drug delivery, improving the stability and efficiency of drugs, stabilizing bioactive agents by encapsulation or surface anchorage, and controlling drug or biological components release at target sites [6,7].

Tannic acid (TA), which belongs to the subset of hydrolyzable tannin producers, can be found in many different natural sources such as green tea, grapes, wine, and others [8]. TA is capable of functionalizing the surfaces of various organic and inorganic substrates in an aqueous state, due to its abundant pyrogallol and catechol groups [9,10]. TA is commonly used in materials engineering due to its special structural features that facilitate interactions with various substances due to its coordination, electrostatic, hydrogen bonding, and hydrophobic interaction capabilities [11,12,13]. TA has long been used in countless biomedical applications due to its antibacterial, antioxidant, anti-allergic, anti-diabetic, anti-cancer, and anti-inflammatory properties [14,15]. Recently, our group reported that TA-coated PCL scaffolds had superior antioxidant and anti-inflammatory effects compared to uncoated PCL scaffolds [10]. TA, which contains abundant hydroxyl functional groups, can provide the capacity to develop the solubilization, complexation, and encapsulation of therapeutic carriers for long and sustained release applications [16,17,18]. TA, containing plentiful hydrophobic aromatic rings and hydrophilic phenolic hydroxyl groups, is also known to react with various materials by covalent and non-covalent interactions due to its hydrogen bonding, electrostatic, and hydrophobic interplay [19,20]. TA, which includes carbonyl and phenol functional groups, has been shown to be a key component for binding to various biopolymers and biomolecules that rely on hydrogen bonding [21,22]. Because TA can bind to drug molecules primarily via hydrophobic interactions, it has inherently wished nanocarrier properties, which form a self-assembled cross-linked network through the role of a hydrogen-bonding donor [23,24,25]. 

Bisphosphonates (BPs) are extensively used in diseases connected with osteoporosis, bone metastasis, hypercalcemia of malignancy, and Paget’s disease [26]. All BPs have a basic P-C-P structure in the center and two side chains (R1 and R2). BPs are divided into two classes, amino-bisphosphonates (e.g., risedronate, alendronate, ibandronate, zoledronate, and pamidronate) and non-aminobisphosphonates (e.g., clodronate, etidronate, and tiludronate), depending on whether a nitrogen atom is included in a side chain, and their therapeutic effects and mechanisms of action are different. Among the amino-bisphosphonate drugs, alendronate (ALN) is the first commercially valid active molecule for the treatment of osteoporosis [27]. Previous studies reported that ALN not only stimulated the osteogenesis of mouse osteoblastic cells, but also induced the proliferation/differentiation of bone marrow mesenchymal stem cells and adipose-derived stem cells [28,29,30]. These results imply that ALN plays a leading role during cell proliferation and differentiation. Recent studies have reported that the localized and controlled delivery of ALN combined with various scaffolds is advantageous in enhancing bone regeneration [31,32,33]. 

Our ultimate goal was to develop TA-ALNs to improve their antioxidant, anti-inflammatory, and osteogenic potency. Then, we investigated the in vitro antioxidant and anti-inflammatory effects and osteogenic potency of the NPXs containing TA and ALN in osteoblast-like cells.

## 2. Materials and Methods

### 2.1. Preparation of Tannic Acid (TA)-Alendronate (ALN) Nanocomplexes 

Tannic acid (TA, Tokyo Chemical Industry Co., Ltd., Tokyo, Japan)-alendronate (ALN, Tokyo Chemical Industry Co., Ltd., Tokyo, Japan) nanocomplexes were prepared via self-assembly [24,34,35] as follows. To start, TA (0.5 mg) was dissolved in 4-mL vials containing 1 mL of double deionized water (DDI), and different concentrations of ALN (0.05, 0.25, and 0.5 mg) were added into solution mentioned above. The resultant TA-ALN solutions were treated with a probe sonicator (KFS-250 N, power: 45 W, Korea Process Technology Co., Ltd., Seoul, Korea) for 5 min on an ice bath. Then, the different TA-ALN solutions were constantly stirred with a magnetic ball at 300 rpm for 24 h. Various TA-ALN solutions were individually cleaned by dialysis (MWCO: 12,000~14,000, Spectrum Laboratories Inc., Rancho Dominguez, CA, USA) using DDI to remove persistent TA and ALN. The final TA-ALN solutions were freeze-dried (IlShinBioBase Co., Ltd. Gyeonggido, Korea). The different TA-ALNs thus produced are henceforward referred to as TA-ALN (1:0.1), TA-ALN (1:0.5), and TA-ALN (1:1).

### 2.2. Physicochemical Characterization 

To investigate the morphology, TA-ALN (1:0.1), TA-ALN (1:0.5), and TA-ALN (1:1) were dispersed in ethanol (C_2_H_5_OH, DUKSAN, Ansan, Korea) using a probe sonicator. Each NPX type was carefully shifted to a cover glass, then dried in a drying oven to remove any remaining ethanol. After that, each TA-ALN sample was shifted to the transmission electron microscope (TEM) dish, and then their morphologies were examined with the FE-TEM (JEM-F200, JEOL Ltd., Tokyo, Japan). The particle sizes (Z-average diameter, nm), the size distributions, and the polydispersity index (PDI) values of the various TA-ALNs were investigated using a Zetasizer device (Malvern Instruments, Malvern, UK) on the basis of the dynamic light scattering (DLS) principle. The zeta-potentials (mV) of the various TA-ALNs were determined using a laser Doppler electrophoretic procedure with a Zetasizer. To perform each measurement, each TA-ALN sample (100 µg) was added to 10 mL of DDI, followed by probe sonication for 30 s over ice. Next, each dispersed sample was situated in a disposable cuvette and then analyzed with a Zetasizer device. Each measurement was reported from 3 readings. The stretching frequencies of the various TA-ALNs were determined by ATR-FTIR spectrometer (Thermo Electron Corp., Madison, WI, USA) at the scales of 4000 and 400 cm^−1^ at a 4 cm^−1^ resolution. To determine the total phenolic content of the TA-ALN, we used the Folin-Ciocalteu method [36]. In brief, each TA-ALN (1 mg/mL) was mixed with 750 µL of Folin-Ciocalteu reagent and allowed to react at room temperature (RT) for 1 min. After reaction, 750 µL of sodium bicarbonate (Na_2_CO_3_, 5% [*w/v*]) solution was added to the blended solution, followed by incubation for 1 h in the dark. The absorbance was monitored at 765 nm using a Multimode Reader (Thermo Fisher Scientific, Waltham, MA, USA). The phenolic concentration was determined by comparison with the standard curve of gallic acid with a standard curve equation (Y = 0.0177X − 0.0352, R^2^ = 0.9965). The total phenolic contents of the samples were expressed in terms of gallic acid equivalents (mg/L).

### 2.3. In Vitro Cytotoxicity Examination of Each TA-ALN 

Cytotoxicity examinations of each TA-ALN were conducted with a cell counting kit (CCK-8; Cell Counting Kit-8, Dojindo, Inc., Rockville, MD, USA). MC3T3-E1 cells (Korean Cell Line Bank, Seoul, Korea) were plated in 96-well culture plates at 1 × 10^4^ cells/well, followed by incubation with DMEM (Thermo Fisher Scientific) including 10% FBS (Thermo Fisher Scientific) and 1% antibiotics (Thermo Fisher Scientific) in an incubator at 37 °C with 5% CO_2_ for 24 h. After the incubation for 24 h, the cells were exposed to various TA-ALNs at a concentration of 50 µg/mL. After exposure for 24 or 48 h, CCK-8 reagent was added to each cell and followed by further incubation for 1 h at 37 °C in the dark. The absorbance was recorded at 450 nm using a multimode reader. Cells cultivated without any TA-ALN treatment were used as a control group. All experiments per time period were repeated three times.

### 2.4. Antioxidant Effects

#### 2.4.1. Total Antioxidant Potential

We measured the 2,2-diphenyl-1-picrylhydrazyl (DPPH) scavenging activity of each type of TA-ALN. Each TA-ALN type (50 µg/mL) was placed in DDI at 37 °C for 24 h. After incubation, the extract solution (200 µL) from each TA-ALN type was blended with 800 µL of DPPH (0.3 mM) dissolved in CH_3_OH, then vortexed thoroughly and reacted at RT in the dark for 30 min. The absorbance of the supernatant of the final solution obtained through centrifugation was measured at 517 nm using a multimode reader. 

#### 2.4.2. In Vitro Reactive Oxygen Species (ROS) Scavenging Capacity of TA-ALN at Cellular Levels

To investigate and compare the ROS scavenging capacities of the different TA-ALNs at the cellular level, we used qualitative and quantitative methods such as 2′,7-dichlorodihydrofluorescein diacetate (DCFDA) staining. For this study, cells (1 × 10^4^ cells/well) were seeded on cover glasses into 24-well cell culture plates and incubated for 24 h. After incubation, cells were treated with DMEM with 300 µM H_2_O_2_ at 37 °C for 30 min, and this was followed by the addition of the extract from each TA-ALN and 24 h of treatment. After treatment for 24 h, cells were stained with DCFDA (25 µM) in the dark for 30 min, rinsed with PBS, and fixed with 3.7% paraformaldehyde for 30 min.

#### 2.4.3. Defense of Cell Viability under High-ROS Conditions

To confirm whether TA-ALN can defend the viability of cells under high-ROS conditions, cells (1 × 10^5^ cells/well) were seeded into 24-well cell culture plates and cultured with DMEM containing each TA-ALN (50 μg/mL). After incubation for 24 h, cells were exposed to serum-free DMEM containing 300 µM H_2_O_2_ at 37 °C for 30 min and were further cultured with DMEM for 24 h. Then, CCK-8 reagent was added to cells, followed by incubation at 37 °C for 1 h. The supernatant of the cells was transferred to 96-well plates and the absorbance was recorded using a multimode reader at a wavelength of 450 nm. Wells that were not exposed to H_2_O_2_ were regarded as the negative control.

### 2.5. Anti-Inflammatory Ability of TA-ALN

To assess the anti-inflammatory abilities of TA-ALN, cells (1 × 10^5^ cells/well) were seeded on 24-well cell culture plates containing DMEM, incubated for 24 h, and then treated with lipopolysaccharide (LPS, 100 ng/mL) in the presence or absence of each TA-ALN (50 µg/mL). After treatments for 3 days, RNA was acquired from the cultured cells using a RNeasy Plus mini kit (Qiagen, Valencia, CA, USA) according to the manufacturer’s instructions. Total RNA (1 µg) after the RNA density investigation was a reverse-transcription of cDNA using AccuPower RT PreMix (Bioneer, Daejeon, Korea). The sequences of the pro-inflammatory mediator gene primers are provided in the Supplementary Materials (Table S1). The ABI7300 Thermal Cycler using the SYBR Green PCR master mix (Applied Biosystems, Foster City, CA, USA) was utilized to perform real-time polymerase chain reaction (real-time PCR). Pro-inflammatory mediator genes were normalized using glyceraldehyde 3-phophate dehydrogenase (GAPDH). We used four samples per type of TA-ALN.

### 2.6. Early Osteogenesis Quantitative Assessment

To estimate alkaline phosphatase (ALP) levels as an early osteogenesis quantitative measurement, cells (1 × 10^5^ cells/well) were cultivated on 24-well cell culture plates, and then the cultured cells were treated with TA-ALN (50 µg/mL). After being processed for 3, 7, or 9 days, cells were cleaned with phosphate-buffered saline (PBS, pH 7.4, Welgene Inc., Gyeongsan-si, Gyeongsangbuk-do, Korea), and 1× RIPA buffer (lysis buffer) was added to each cell for cell lysis. Then, the lysed cells were carefully transferred to e-tubes and centrifuged at 13,500 rpm for 10 min, and *P*-nitrophenyl phosphate (Sigma-Aldrich, St. Louis, MO, USA) was added to the cell lysates. The resultant solution was incubated at 37 °C for 30 min. The process was maintained by the supplementation of 1 N NaOH solution. Protein concentrations were measured by both the Bradford reagent (Bio-Rad Laboratories, Inc., Hercules, CA, USA) and bovine serum albumin (BSA, Bio-Rad Laboratories, Inc.) as a standard. The absorbance was determined by a multimode reader at a wavelength of 405 nm. All experiments were repeated three times at 3, 7, or 9 days. We used four samples per type of TA-ALN.

### 2.7. Late Osteogenesis Quantitative Assessment

To verify the late osteogenesis quantitative measurement, we evaluated the calcium contents at 7 or 21 days. Cells (1 × 10^5^ cells/well) were inoculated on 24-well cell culture plates, and then the seeded cells were exposed each TA-ALN (50 µg/mL). At 7 or 21 days, 0.5 N hydrochloric acid solution was added to cells, which were incubated with stirring at 37 °C for overnight. After incubation, cells were exposed to a blending solution including calcium standard solution and reagent solutions containing both o-cresolphthalein complexone and 8-hydroxy-quindine for 1 min. Then, 2-amino-2-methyl-1-propanol (20 µL, Sigma-Aldrich) buffer were added to the mixing solution. The combined solution was reacted for 15 min. The final solution was moved to 96-well plates, and calcium contents were quantitated using absorbance with a multimode reader at 575 nm. All experiments were repeated three times at 7 or 21 days. We used four samples per type of TA-ALN.

### 2.8. Assays for Osteogenesis-Related Genes

To confirm the osteogenic potency of the TA-ALN, the mRNA levels of osteogenesis detailed genes such as collagen type I (COL1A1) and osteocalcin (OCN) were quantitatively determined using real-time PCR. Cells at a concentration of 1 × 10^5^ cells/well were seeded on 24-well cell culture plates and exposed to one of the three types of NPXs (50 µg/mL). After treatment for 21 days, an RNeasy Plus mini kit was utilized to acquire ribonucleic acid (RNA) in the harvested cells. Next, we measured the total RNA levels and generated reverse-transcription of cDNA from total RNA (1 µg) using AccuPower RT PreMix. The primer sequences used for the real-time PCR are listed in the Supplementary Materials (Table S2). Real-time PCR was conducted on an ABI7300 Thermal Cycler (Applied Biosystems, Foster, CA, USA) using the SYBR Green PCR master mix. Osteogenesis-related genes were normalized using GAPDH. We used 4 samples per type of TA-ALN.

### 2.9. Statistical Analysis 

All the data were presented as the means ± standard deviations. Statistical analysis was performed by one-way ANOVA with post hoc Holm-Sidak test in SigmaPlot (Systat Software, Inc., San Jose, CA, USA). *p*-values were compared among all TA-ALNs. *p*-values less than 0.05 or less than 0.01 were considered significant.

## 3. Results

### 3.1. Characterization of TA-ALN

**Figure 1:**
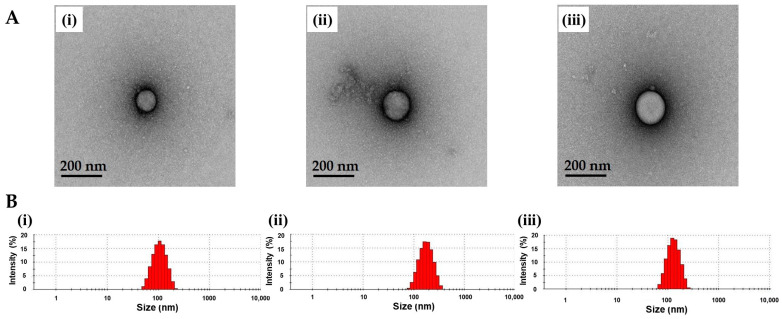
(**A**) TEM images of (**i**) TA-ALN (1:0.1), (**ii**) TA-ALN (1:0.5), and (**iii**) TA-ALN (1:1). (**B**) Size distribution curves of (**i**) TA-ALN (1:0.1), (**ii**) TA-ALN (1:0.5), and (**iii**) TA-ALN (1:1).

TA-ALN were fabricated by self-assembly based on hydrogen bonding [13,24]. The forms of three types of TA-ALN were examined by TEM (Figure 1A). Each TA-ALN showed spherical in shape. The hydrodynamic diameters of the three types of TA-ALNs obtained from DLS analysis ranged from approximately 108 to 180 nm, and their PDIs were between 0.253 and 0.278 (Figure 1B). The verified zeta-potential values of the TA-ALN (1:1) (−2.84 ± 1.55 mV) appeared obviously greater compared to those of TA-ALN (1:0.1) (−15.57 ± 1.64 mV) or the TA-ALN (1:0.5) (−13.60 ± 0.21 mV) due to increasing the amount of ALN from 0.05 mg to 0.5 mg. Thus, these results indicated the successful preparation of TA-ALNs.

**Figure 2:**
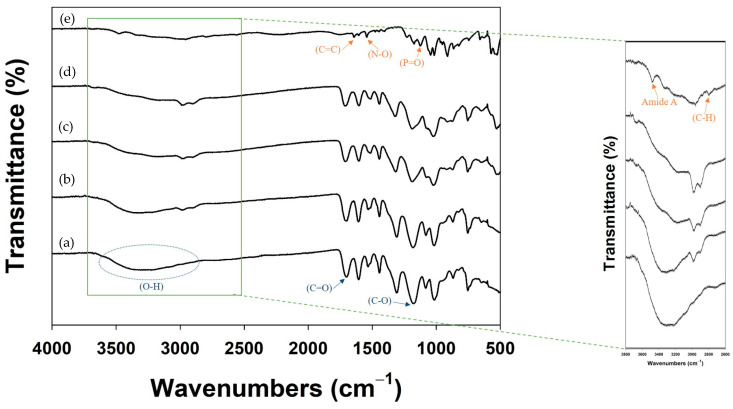
FT-IR spectra of (**a**) TA, (**b**) TA-ALN (1:0.1), (**c**) TA-ALN (1:0.5), (**d**) TA-ALN (1:1), and (**e**) ALN. Magnified FT-IR spectra range from 3800 to 2600 cm^−1^.

Next, the formation of each TA-ALN was assessed with ATR-FTIR analysis (Figure 2). In the ATR-FTIR spectra, TA showed major absorption peaks at 3200–3550 cm^−1^ (phenolic O–H stretching), 1714 cm^−1^ (carbonyl C=O stretching), and 1205 cm^−1^ (aromatic C–O stretching). The characteristic peaks for ALN were at 3200–3550 cm^−1^ (phenol O–H stretching), 3260 cm^−1^ (amide A, two N-H stretching absorptions), 2950 cm^−1^ (alkyl C–H stretching), and 1200–1500 cm^−1^ (P=O stretching vibration). After nanocomplexing, the FT-IR spectra of the TA-ALN (1:0.1) and TA-ALN (1:0.5) showed a wide and strong absorption band around 3380 cm^−1^ and a weak band at 2800–3000 cm^−1^, which correspond to the –OH stretching mode (hydrogen bonding) and C-H stretching vibrations, respectively. Moreover, the FT-IR of the TA-ALN (1:1) displayed the peak of the amide I shifted to about 3700 cm^−1^, and the powerful band at 2800–3000 cm^−1^ was ascribed to C–H stretching vibrations. These results suggested that the interaction between TA and ALN consisted of not only hydrogen bonds, but also the effects of the increasing exposure of amine-ALN. The total phenolic content in each TA-ALN was deduced using gallic acid (GA) as a standard. The total phenolic contents were 32.93 ± 0.40 μg for TA-ALN (1:0.1), 27.63 ± 0.09 μg for TA-ALN (1:0.5), and 21.04 ± 0.04 μg for TA-ALN (1:1).

### 3.2. Cytotoxic Results of TA-ALN on MC3T3-E1 Cells

To determine the cytotoxicity of each TA-ALN in MC3T3-E1 cells, CCK-8 assays were assessed at 24 or 48 h after each TA-ALN treatment. As shown in Figure S1, the survival abilities of MC3T3-E1 cells after exposure to each type of TA-ALN for 48 h showed more than 95% of that in the control group. Based on the results of the cell viability analysis, none of the TA-ALN types created any clear cytotoxicity in the MC3T3-E1 cells. 

### 3.3. Anti-Oxidant Study

#### 3.3.1. Antioxidant Investigation of TA-ALN

**Figure 3:**
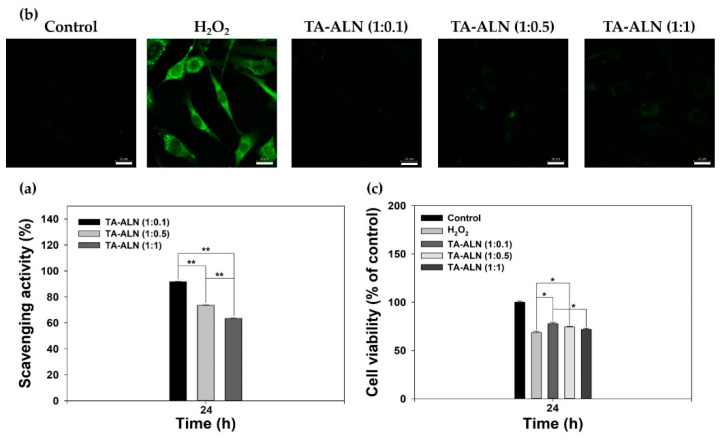
(**a**) Antioxidant capacities of TA-ALN (1:0.1), TA-ALN (1:0.5), and TA-ALN (1:1) were investigated by analyzing DPPH scavenging capacity. (**b**) Representative fluorescence views of intracellular ROS in osteoblast-like cells treated with extracts from TA-ALN (1:0.1), TA-ALN (1:0.5), and TA-ALN (1:1) for 24 h after pretreatment with 300 µM H_2_O_2_ for 30 min. (**c**) Viabilities of cells treated with TA-ALN (1:0.1), TA-ALN (1:0.5), and TA-ALN (1:1) for 24 h after pre-exposure to 300 µM H_2_O_2_. The *p*-value is a comparison between all TA-ALN types. * *p* < 0.05; ** *p* < 0.01; *n* = 4 per group.

The antioxidant capacities of TA-ALN were monitored by DPPH assay [10]. As shown in Figure 3a, the TA-ALN (1:1) were insufficient for DPPH scavenging. However, the TA-ALN (1:0.1) had an extremely high DPPH scavenging rate of about 91% and also had strong antioxidant activity as compared to the TA-ALN (1:0.5) or TA-ALN (1:1). These results indicated that the TA-ALN (1:0.1) had superior antioxidant capacity as well as efficient ROS-scavenging potency compared with the other TA-ALN. Previous studies reported that TA-coated PCL scaffolds or TA-CaCO_3_ materials had much higher DPPH scavenging activities than the PCL scaffolds or CaCO_3_ materials alone [10,37]. These results indicated that TA-based materials had ROS-scavenging activity. 

#### 3.3.2. In Vitro ROS Scavenging and Defense Effects of TA-ALN at the Cellular Level

Hydrogen peroxide (H_2_O_2_), which has powerful oxidizing qualities, is generally used as an inducer of oxidative stress. Furthermore, cells treated with exogenous hydrogen peroxide exhibit intracellular ROS formation [38,39]. To confirm the antioxidant capacities of each TA-ALN at the cellular level, MC3T3-E1 cells were pre-treated with H_2_O_2_ (300 μM) to induce oxidative stress, followed by exposure to extracts from the TA-ALN for 24 h, and the intracellular ROS levels after treatment with each TA-ALN were observed from the fluorescence strength of DCFDA using CLSM. As shown in Figure 3b, normal cells (no treatment with H_2_O_2_) were observed to have no fluorescence, whereas cells treated with H_2_O_2_ exhibited powerful fluorescence. However, the treatments with the extracts including different TA-ALNs revealed a noticeable reduction in fluorescence strength, suggestive of the outstanding ROS scavenging capacities of the NPXs in cells. 

Previous studies have reported that H_2_O_2_ itself is not reactive but can be toxic to cells because it can induce hydroxyl groups in cells [38,39]. To further evaluate the ROS scavenging potencies of TA-ALN, we investigated the proliferation of cells treated with each TA-ALN type against the H_2_O_2_ challenge for 24 h. As shown in Figure 3c, compared with the control group, the viability of cells treated with 300 µM H_2_O_2_ demonstrated a notable reduction due to the oxidative impairment to cellular parts [40]. However, the viabilities of cells cultured with the TA-ALN markedly advanced in an ALN low concentration-dependent fashion were much higher than that of H_2_O_2_ situation (* *p* < 0.05). Coincidental with our previous results, TA-ALN effectively protected the cells against high-ROS conditions [10,37,41].

### 3.4. Anti-Inflammatory Activities of TA-ALN in Inflamed MC3T3-E1 Cells 

**Figure 4:**
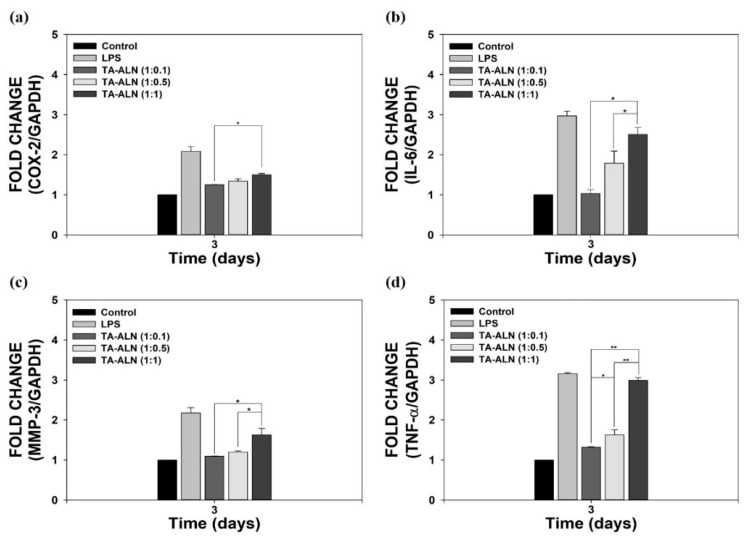
In vitro anti-inflammatory capacities of TA-ALN in inflamed osteoblast-like cells. mRNA levels of pro-inflammatory mediators confirmed by real-time PCR: (**a**) COX-2, (**b**) IL-6, (**c**) MMP-3, and (**d**) TNF-α in LPS-promoted MC3T3-E1 cells on days 3 after treatment with each TA-ALN. The *p*-value is a comparison between all TA-ALN types. * *p* < 0.05; ** *p* < 0.01; GAPDH, glyceraldehyde 3-phophate dehydrogenase; *n* = 4 per group.

To assess the anti-inflammatory powers of TA-ALN, we measured the mRNA levels of pro-inflammatory mediators such as COX-2, IL-6, MMP-3, and TNF-α in LPS-induced MC3T3-E1 cells, because LPS induction can overexpress these pro-inflammatory markers [10,37,42]. As shown in Figure 4, compared with cells without LPS induction, the promoted pro-inflammatory mediators were evident in LPS-induced cells for 3 days. However, the mRNA levels of pro-inflammatory mediators in cells treated with TA-ALN (1:1) were slightly decreased, whereas the expression levels in the cells decreased significantly in the cells treated with TA-ALN (1:0.5) or TA-ALN (1:0.1). The mRNA levels of pro-inflammatory mediators in the LPS-induced cells treated with TA-ALN (1:0.1) were markedly suppressed compared with those of all other groups. Coincidental with previous results, we also demonstrated that TA-based materials effectively suppressed the expressions of pro-inflammatory mediators in LPS-induced MC3T3-E1 cells, indicating that TA-ALNs are effective materials to prevent inflammation reactions [10,37,43]. 

### 3.5. Early and Late Osteogenesis Quantitative Assessment 

**Figure 5:**
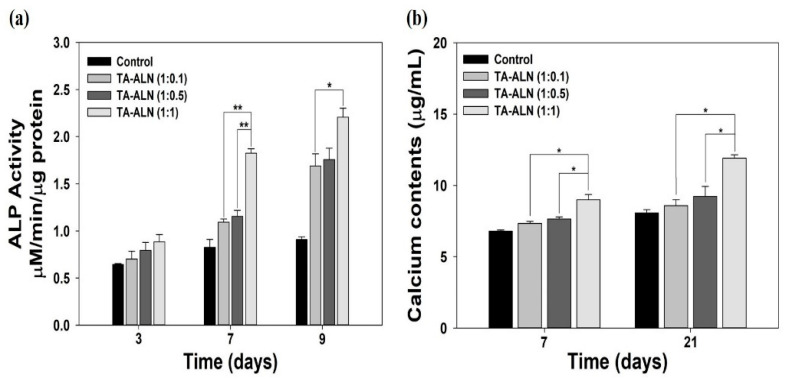
Early and late osteogenesis assessments. (**a**) ALP activity of MC3T3-E1 cells cultured with TA-ALN (1:0.1), TA-ALN (1:0.5), and TA-ALN (1:1) after 3, 7, and 9 days of culture. (**b**) Calcium accumulation in MC3T3-E1 cells cultivated with TA-ALN (1:0.1), TA-ALN (1:0.5) and TA-ALN (1:1) after 7 and 21 days. The *p*-value is a comparison between all TA-ALN types. * *p* < 0.05; ** *p* < 0.01; *n* = 4 per group.

To qualitatively and quantitatively verify the early osteogenesis differentiation tracker, the ALP activities of MC3T3-E1 cells were investigated on days 3, 7, and 9 after osteogenic stimulation. ALP, a well-known enzyme associated with cell membranes, previously exhibited early expression coincident with osteoblast differentiation. Moreover, ALP has been extensively recommended as an early osteoblast differentiation tracker [31,32,33]. As shown in Figure 5a, the ALP activities of cells treated with each TA-ALN developed gradually in a time-dependent manner. On day 3, there were no significant differences in the ALP activities of cells in any TA-ALN groups. At seven days, the ALP activity of cells treated with the TA-ALN (1:1) was significantly higher than that of those of treated with the TA-ALN (1:0.1) or TA-ALN (1:0.5) (** *p* < 0.01). After 9 days, a significant difference in the ALP levels of cells was evident between those treated with the TA-ALN (1:1) and the TA-ALN (1:0.1) (* *p* < 0.05). However, a significant difference was not shown in the ALP activity between cells treated with the TA-ALN (1:0.5) and those treated with the TA-ALN (1:1) at nine days. Consistent with our previous studies, the current study also demonstrates the early osteogenesis effects of TA-ALN in a dose-dependent manner [31,44]. 

For the quantitative evaluation of the late osteogenesis, the quantities of calcium collected by MC3T3-E1 cells treated with each TA-ALN were determined after 7 days and 21 days of incubation. Calcium contents have been generally shown to be associated with a late osteoblast differentiation marker [32,33,44]. As shown in Figure 5b, the calcium collection in the MC3T3-E1 cells cultivated with the TA-ALN (1:1) was notably greater than that in the MC3T3-E1 cells grown with the TA-ALN (1:0.1) or TA-ALN (1:0.5) on day seven (* *p* < 0.05). On day 21, important differences were noticed in the total quantity of calcium accumulated by the cells incubated with the TA-ALN (1:1) and TA-ALN (1:0.1) and between those cultured with the TA-ALN (1:1) and TA-ALN (1:0.5) (* *p* < 0.05). Previous studies reported that treatment with ALN joined with various types of specimens, such as ceramic scaffolds, porous microspheres, 3D printed scaffolds, chitosan scaffolds, and nanofibrous scaffolds, promoted calcium aggregation [31,44,45]. These previous results were coincidental with our results, indicating that TA-ALN promoted calcium collection in cells in a dose-dependent manner associated with ALN. 

### 3.6. Measurement of Osteogenesis-Specific Genes 

**Figure 6:**
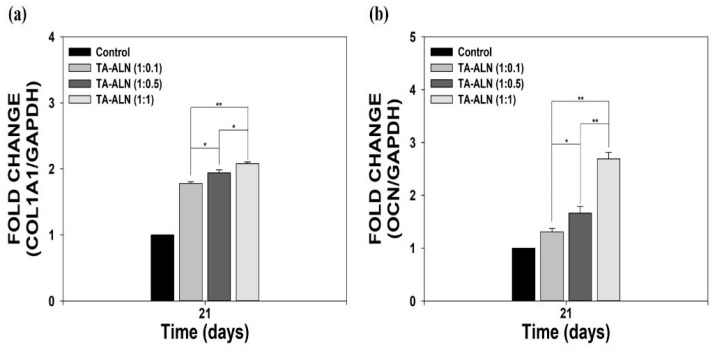
The mRNA levels of osteogenesis-related genes, including (**a**) COL1A1 and (**b**) OCN in MC3T3-E1 cells treated with TA-ALN (1:0.1), TA-ALN (1:0.5), and TA-ALN (1:1) after 21 days of incubation. The *p*-value is a comparison between all TA-ALN types. * *p* < 0.05; ** *p* < 0.01; *n* = 4 per group.

To further assess the osteogenesis benefits of TA-ALN, we investigated the osteogenesis-specific genes including COL1A1 and OCN via real-time PCR after 21 days of incubation. The organic part of the bone extracellular matrix (ECM) consists of collagen type I (COL1A1) and also contains non-collagen bone proteins, including osteopontin (OPN) and osteocalcin (OCN), which are important for regulating the organic matrix and bone mineralization [8,33,46]. OCN and COL1A1 genes play important roles in regulating osteoblast differentiation as well as being used as a serum marker for bone formation [8,41,46]. As shown in Figure 6, the COL1A1 and OCN gene expression levels in the cells treated with the TA-ALN increased notably in an ALN concentration-dependent manner. In addition, the expressions of the COL1A1 and OCN genes in the cells cultured with the TA-ALN (1:1) were significantly greater than those in cells grown with the TA-ALN (1:0.1) or TA-ALN (1:0.5). Consistent with our previous reports that the substances containing alendronate could increase osteogenic-related marker genes during the osteoblast differentiation of cells [33,44,45,47], TA-ALN effectively increased the expression levels of osteogenesis-specific genes in cells. This osteogenesis effect might be related to the effective osteogenic capacity of the ALN molecules in the TA-ALN. 

## 4. Conclusions

In the present study, TA-ALNs with different ALN concentrations were prepared by self-assembly based on hydrogen bonding. We examined the capacities of the TA-ALNs for antioxidant, anti-inflammatory, and osteogenic potency in MC3T3-E1 cells. TA-ALNs not only effectively scavenged ROS in cells, but also protected cells from the high-ROS conditions and can markedly decrease the expression of pro-inflammatory mediators in LPS-induced cells, owing to the antioxidant and anti-inflammatory properties of the TA molecules within the TA-ALN. Moreover, the TA-ALN improved the osteogenic potencies of MC3T3-E1 cells by markedly promoting ALP activity and calcium collection in an ALN concentration-dependent manner. Furthermore, the TA-ALN induced the expressions of late osteogenesis-specific genes (COL1A1 and OCN) in an ALN concentration-dependent fashion. Therefore, these TA-ALNs may be used to advance bone repair and regeneration in bone diseases, disorders, fractures, and defects. Meanwhile, our study did not evaluate the stability of the TA-ALN nanocomplexes over time. However, their stability issues would be resolved by coating of TA-ALNs on the surfaces of scaffolds or embedding them into a matrix to further apply for bone tissue regeneration. 

## Data Availability

The data presented in this study are available in supplementary materials.

## Supplementary Materials

The following are available online at https://www.mdpi.com/article/10.3390/nano11071812/s1, Figure S1: Outcome of cytotoxicity test of TA-ALN (1:0.1), TA-ALN (1:0.5) and TA-ALN (1:1) on MC3T3-E1 cells 24 and 48 h, Table S1: Real-time PCR primer sequences for genes connected to the pro-inflammatory mediators, Table S2: Real-time PCR primer sequences for genes related to the osteogenesis factors.
