# Supplementary material for: Multifunctional Tannic Acid-Alendronate Nanocomplexes with Antioxidant, Anti-Inflammatory, and Osteogenic Potency
Source: Nanomaterials (Basel). 2021 Jul 13;11(7):1812. doi: 10.3390/nano11071812 (PMC8308329; doi:10.3390/nano11071812)
Supplement: Supplementary file 1 [file nanomaterials-11-01812-s001.zip › nanomaterials-1284778-supplementary.pdf]

Supplementary Materials

Multifunctional Tannic Acid-Alendronate Nanocomplexes with Antioxidant, Anti-Inflammatory, and Osteogenic Potency

Somang Choi [1], Han-Saem Jo [1], Heegyeong Song [2], Hak-Jun Kim [1], Jong-Keon Oh [1], Jae-Woo Cho [1,*], Kyeongsoon Park [2,*] and Sung-Eun Kim [1,*]

[1] Department of Orthopedic Surgery and Nano-Based Disease Control Institute, Korea University Guro Hospital, #148, Gurodong-ro, Guro-gu, Seoul 08308, Republic of Korea; chlthakd1029@naver.com (S.C.); luchiatkfkd@naver.com (H.-S.J.); dakjul@korea.ac.kr (H.-J.K.); jkoh@korea.ac.kr (J.-K.O.)

[2] Department of Systems Biotechnology, Chung-Ang University, Anseong 17546, Korea; island6231@gmail.com

* Correspondence: jaewoocho@korea.ac.kr (J.-W.C.); kspark1223@cau.ac.kr (K.P.); sekim10@korea.ac.kr (S.-E.K.); Tel.: +82-2-2626-1869 (J.-W.C.); +82-31-670-3357 (K.P.); +82-2-6738-4514 (S.-E.K.)

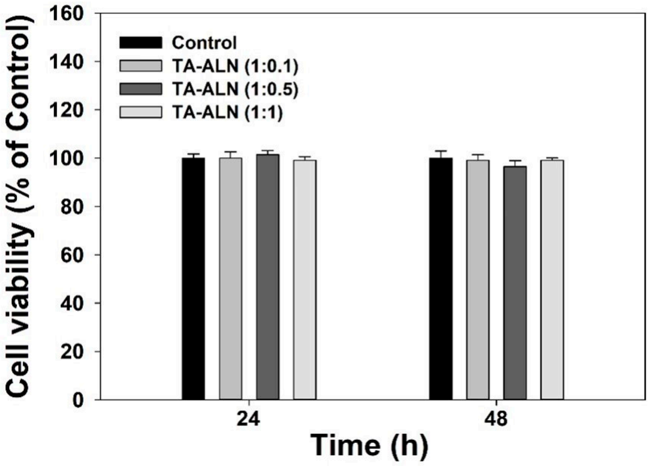

Figure S1. Outcome of cytotoxicity test of TA-ALN (1:0.1), TA-ALN (1:0.5) and TA-ALN (1:1) on MC3T3-E1 cells 24 and 48 h (n = 4 per group).

Table S1. Real-time PCR primer sequences for genes connected to the pro-inflammatory mediators

Gene	Primer (forward)	Primer (reverse)
COX-2	TAT CAG GTC ATC GGT GGA GA	CGA AGC CAG ATG GTA GCA TA
IL-6	TAC CAC TTC ACA AGT CGG AGG C	CTG CAA GTG CAT CAT CGT TGT TC
MMP-3	CTC TGG AAC CTG AGA CAT CAC C	AGG AGT CCT GAG AGA TTT GCG C
TNF-α	GGT GCC TAT GTC TCA GCC TCT T	GCC ATA GAA CTG ATG AGA GGG AG

Table S2. Real-time PCR primer sequences for genes related to the osteogenesis factors

Gene	Primer (forward)	Primer (reverse)
OCN	GCT GCT ACT GTG GAA GAG AAG G	CCA CAG ATG ACT GAG AAC GCC T
COL1A1	CAA GAG TGG TGA TCG TGG TG	GCC TGT CTC ACC CTT GTC A
